# ShiF acts as an auxiliary factor of aerobactin secretion in meningitis *Escherichia coli* strain S88

**DOI:** 10.1186/s12866-019-1677-2

**Published:** 2019-12-17

**Authors:** Mathieu Genuini, Philippe Bidet, Jean-François Benoist, Dimitri Schlemmer, Chloé Lemaitre, André Birgy, Stéphane Bonacorsi

**Affiliations:** 1 University Paris Diderot, Sorbonne Paris Cité, IAME, F-75018 Paris, France; 2 Service de microbiologie, Hôpital Robert-Debré, AP-HP, 48 Bd Sérurier, 75019 Paris, France; 3 Service de Biochimie-Hormonologie, AP-HP Hôpital Robert Debré, Paris, France; 4 University Paris-Sud, Chatenay-Malabry, France

**Keywords:** *Escherichia coli*, Siderophore, Aerobactin, ShiF

## Abstract

**Background:**

The neonatal meningitis *E. coli* (NMEC) strain S88 carries a ColV plasmid named pS88 which is involved in meningeal virulence. Transcriptional analysis of pS88 in human serum revealed a strong upregulation of an ORF of unknown function: *shiF,* which is adjacent to the operon encoding the siderophore aerobactin. The aim of this work is to investigate the role of *shiF* in aerobactin production in strain S88.

**Results:**

Study of the prevalence of *shiF* and aerobactin operon in a collection of 100 extra-intestinal pathogenic *E. coli* strains (ExPEC) and 50 whole genome-sequenced *E. coli* strains revealed the colocalization of these two genes for 98% of the aerobactin positive strains. We used Datsenko and Wanner’s method to delete *shiF* in two S88 mutants. A cross-feeding assay showed that these mutants were able to excrete aerobactin meaning that *shiF* is dispensable for aerobactin excretion. Our growth assays revealed that the *shiF*-deleted mutants grew significantly slower than the wild-type strain S88 in iron-depleted medium with a decrease of maximum growth rates of 23 and 28% (*p* < 0.05). Using Liquid Chromatography-Mass Spectrometry, we identified and quantified siderophores in the supernatants of S88 and its *shiF* deleted mutants after growth in iron-depleted medium and found that these mutants secreted significantly less aerobactin than S88 (− 52% and - 49%, *p* < 0.001).

**Conclusions:**

*ShiF* is physically and functionally linked to aerobactin. It provides an advantage to *E. coli* S88 under iron-limiting conditions by increasing aerobactin secretion and may thus act as an auxiliary virulence factor.

## Background

The neonatal meningitis *E. coli* (NMEC) strain S88 carries a ColV plasmid named pS88 which is involved in meningeal virulence [1, 2]. We have previously performed a transcriptional analysis of pS88 in human serum to improve comprehension of virulence determinants of pS88 in neonatal meningitis. This study revealed a strong transcription upregulation for all iron uptake systems, including the well-known siderophores salmochelin and aerobactin, but also for two genes with unknown function: *ssbL* and *shiF* [3]. We have previously described the role of *ssbL*, the most strongly upregulated gene in these conditions and showed that it acts as an auxiliary virulence factor by boosting the catecholate and phenolate siderophores (yersiniabactin and salmochelin) production through the shikimate pathway [4].

In this work, we focused on *shiF* which was first described in a pathogenic island named SHI-2 in a *Shigella flexneri* strain [5]. In this strain, as in S88, s*hiF* is adjacent to aerobactin operon and shares the same Fur box consensus sequence and promoting region [3]. Its amino-acid sequence shares a strong (86%) similarity with a membrane protein belonging to the Major Facilitator Superfamily (MFS) but *shiF* as yet no known function.

The aim of this work is to investigate the role of *shiF* in aerobactin production in strain S88 in order to get insight the iron metabolism of extra-intestinal pathogenic *E. coli* strains (ExPEC).

## Results

### The *shiF* gene is strongly linked to aerobactin operon in human ExPEC strains

In order to study the physical link between *shiF* and the aerobactin operon, we performed in silico analysis of 50 whole genome sequenced *E. coli* strains and PCR analysis of 100 ExPEC strains from our collection. Aerobactin operon was found in 17 of the 50 sequenced strains and 83 of our 100 strains (34 and 83% respectively). For 98 of the 100 aerobactin positive strains, we noticed the copresence and the colocalization of *shiF* and *iucA*. Commensal strain ED1a is one of the two exceptions. This strain possesses chromosomal aerobactin operon adjacent to *shiF* but *shiF* is disrupted by a 10 kb insert. The other exception strain was isolated from the cerebral spinal fluid of a newborn with iatrogenic meningitis after rachianasthesia. For all the strains lacking aerobactin operon (*n* = 33 + 17) *shiF* was also missing.

### Presence of *shiF* is dispensable for secretion of aerobactin siderophore

In our cross-feeding experiments, comparison of the strain LG1522 growth halo observed around different strains showed no difference between S88 and its *shiF-*deleted mutants (S88, S88∆*shiF*1 and S88∆*shiF*2) (Additional file 1: Figure S1). The iatrogenic NMEC strain which contains aerobactin operon but not *shiF* was also able to secrete aerobactin. Conversely, no growth of LG1522 was observed in strains S88∆pS88 and ED1a, showing their inability to secrete aerobactin.

### Deletion of *shiF* induces a slower growth in iron-depleted medium

We wanted to perform a quantitative assay to analyze more precisely the potential role of *shiF* during growth. S88, S88∆*shiF*1 and S88∆*shiF*2 grew similarly in iron supplemented MM9 and in lysogeny broth (LB) meaning that the lack of *shiF* has no effect on growth of S88 in iron-rich medium.

**Fig. 1:**
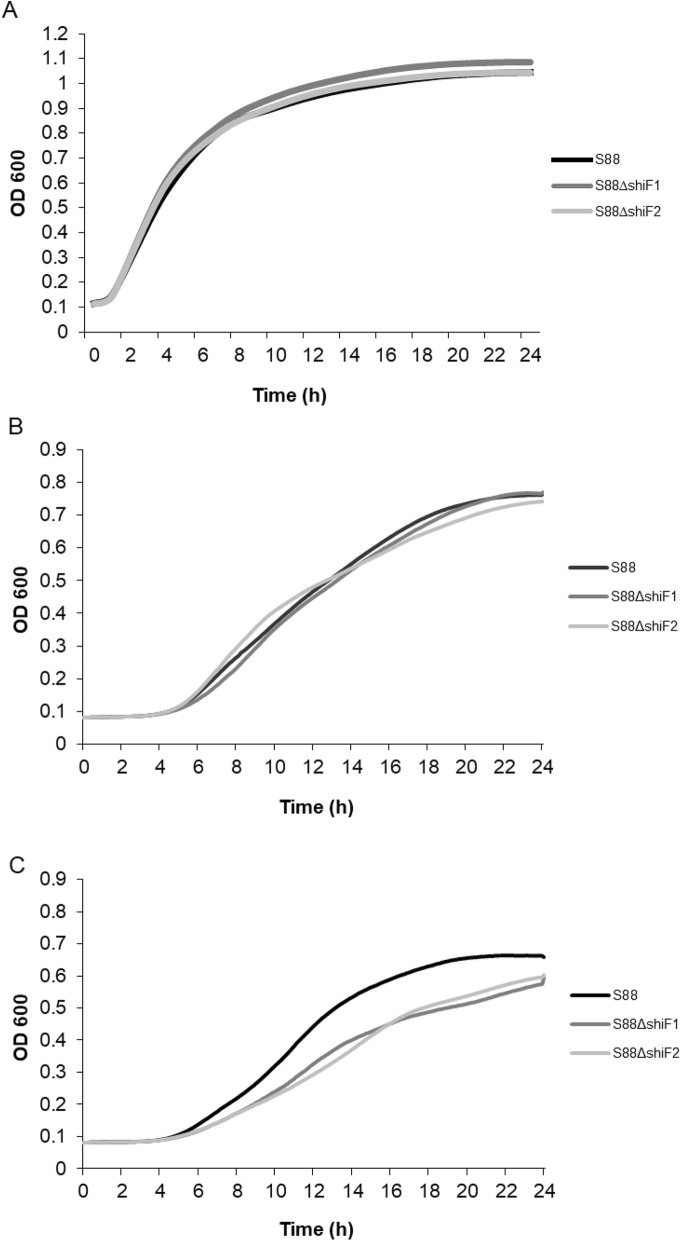
Deletion of *shiF* reduces growth in iron-depleted medium. Strains were grown LB medium (**a**), in MM9 minimal medium with 20 μM iron (**b**) and 100 μM of 2,2′-dipyridyl (**c**). Data presented are average of results from two (**a**) and five (**b** and **c**) independent experiments

In iron-depleted MM9 medium, maximum growth rates showed a significant decrease for both mutants (23 and 28% respectively; *p* < 0.05) and maximum cell densities were 12% less (*p* < 0.01) when compared to the wild type strain S88. No lag between the start of growth was observed between these strains. Growth curves are presented in Fig. 1.

### Deletion of *shiF* decreases aerobactin secretion

**Fig. 2:**
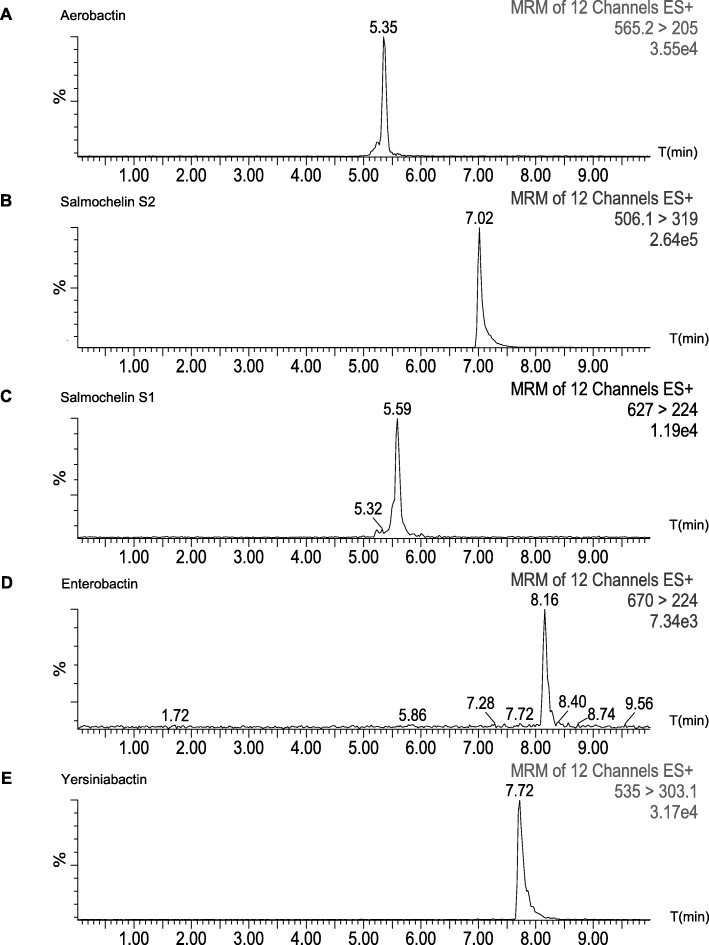
LC-MS/MS siderophore profiles of *E. coli* S88 in iron-limited medium. Aerobactin (**a**), salmochelin S1 (**b**) and S2 (**c**), enterobactin (**d**) and yersiniabactin (**e**) were extracted after addition of 0.1 M ferric chloride and identified with the following specific transitions 565 > 205, 506 > 319, 627 > 224, 670 > 224 and 535 > 303 *m/z* respectively. Data are from the MassLynx software. ES, electrospray positive mode; MRM, multiple reaction monitoring

**Fig. 3:**
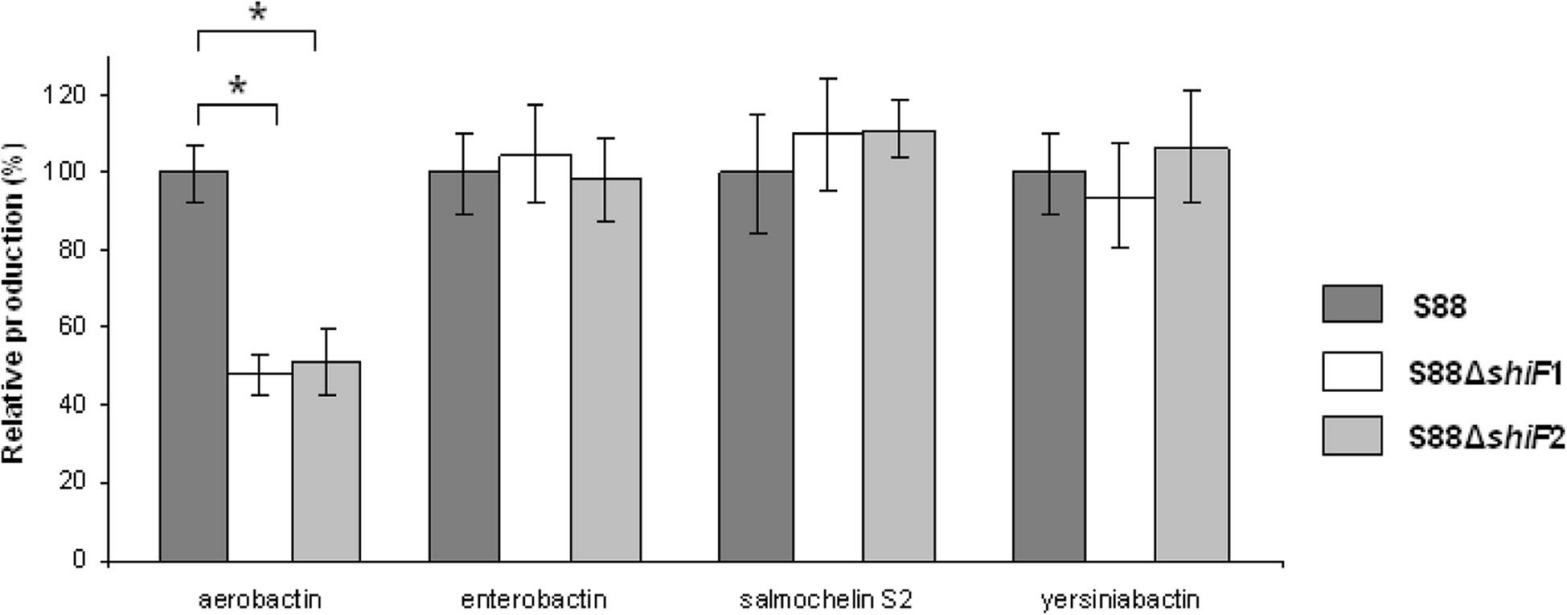
Relative production of aerobactin, enterobactin, salmochelin S2 and yersiniabactin measured by LC-MS/MS. Production of aerobactin, enterobactin, salmochelin S2 and yersiniabactin by the two deleted strains S88∆*shiF*1 and S88∆*shiF*2 were compared to that of the wild type strain S88 in MM9 medium with 100 μM of 2,2′-dipyridyl. Results are expressed in relation to the production of the wild type strain which represent 100%. Data presented are means of 8 independent experiments and were compared using Mann-Whitney’s test. Error bars represent the standard derivations. * *p* < 0.001

We investigated siderophores production by Liquid Chromatography-Mass Spectrometry (LC-MS/MS). We identified specific transitions of aerobactin, enterobactin, salmochelin S1 and S2 and yersiniabactin and confirmed their identity by isotope labeling (Fig. 2).We compared siderophores concentration in culture supernatants of strains grown for 14 h in iron-depleted MM9 medium. Quantification results are shown in Fig. 3. Both *shiF*-deleted strains secreted significantly less aerobactin in their supernatants compared to wild type strain S88 (− 52% and - 49%, *p* < 0.001). There was no significant difference among the strains for production of other siderophores. Intracellular quantification of aerobactin from bacterial pellets showed no significant difference between wild type strain and both *shiF*-deleted mutants (data not shown).

Analyses of strain ED1a showed its ability to produce enterobactin and yersiniabactin but we couldn’t detect any production of aerobactin in accordance with cross-feeding experiment (data not shown).

## Discussion

Iron uptake is essential for *E. coli* growth in urine and blood and siderophores have been shown to play a major role in extra-intestinal virulence [6].

We have previously shown that the transcription of *shiF*, a plasmid located gene contiguous to aerobactin operon in S88 strain, was strongly upregulated during growth in iron depleted environment [3]. As in S88, the sequence analysis of *E. coli* genomes available on data bank revealed a physical link between *shiF* and the aerobactin operon. Here we confirmed this strong physical link in our collection of 100 ExPEC strains. *ShiF* was present and colocalized for 98% of the strains harboring the plasmidic or chromosomic located aerobactin operon. This systematic colocalization and inducibility under low iron condition suggest for *shiF* a role in aerobactin expression and the coacquisition of *shiF* and aerobactin operon was probably guided by a selective advantage.

There are very few data on the role of *shiF*. In a previous work, a cross feeding experiment showed that an *E. coli* strain deleted for *shiF* was able to feed LG1522 which is aerobactin dependent to grow in iron-depleted environment [7]. We did the same assay with our *shiF*-deleted S88 mutants and found the same results. This qualitative experiment led us to conclude that *shiF* is not essential for aerobactin production and/or excretion. However this does not exclude an auxiliary role of *shiF* and we used a quantitative approach to specify the function of *shiF*.

Our growth assays showed that *shiF* was potentially involved in iron metabolism. The *shiF* mutants grew with a significant lower maximum growth rate in an iron-limited minimal MM9 medium. This difference disappeared in iron-rich environment (minimal medium supplemented with iron and LB medium).

In silico analysis of *shiF* sequence reveals a strong similarity (86%) with the membrane proteins belonging to the MFS suggesting a transporter role. This led us to propose two hypothetic functions for *shiF*: a role in aerobactin exportation or a role in aerobactin synthesis by importing lysin which is the aerobactin precursor. We analyzed siderophore production in the supernatant of S88 and S88∆*shiF* in iron-limited medium using LC-MS/MS. There was no difference of enterobactin, yersiniabactin and salmochelin production but we found a significant decrease of aerobactin production for the *shiF*-deleted mutants. This confirmed that *shiF* is not essential but is involved in aerobactin synthesis or exportation. Analysis of intracellular contents of aerobactin showed no significant differences between wild type S88 and mutants but this result can’t lead to conclude on the precise role of *shiF*.

Study of commensal strain ED1a reinforced the functional link between *shiF* and aerobactin. In this strain, *shiF* is adjacent to aerobactin operon but interrupted by a 10 kb insert and probably not functional. Cross-feeding assays and LC-MS/MS both revealed the inability of this strain to produce aerobactin despite the presence of the whole aerobactin operon. The strain harboring aerobactin and a truncated version of *shiF* is non-pathogenic and unable to produce aerobactin. The only NMEC strain harboring aerobactin but not *shiF* was iatrogenically inoculated, thus by-passing the bacteremia step that requires functional iron capture systems.

A limitation of this work was our inability to study our complemented *shiF*-deleted mutant because of its instability in minimal medium. Therefore, we studied two distinct mutants obtained from two separate mutagenesis experiments to reduce the risk of bias due to unwanted genetic events. Both mutants showed the same phenotype. Moreover, since our deletion primers were located inside *shiF* sequence and 32 pb away from the start of aerobactin promoter a polar effect was unlikely [8] .

## Conclusions

*ShiF* is physically and functionally linked to aerobactin. It procures an advantage during growth of *E. coli* S88 under limited iron conditions by acting as a booster for aerobactin secretion. However, we can’t conclude on its precise role. *ShiF* like *ssbL* appears to be an auxiliary virulence factor. This study reinforce the concept that virulence factors probably need acquisition of auxiliary genes to optimize their expression [4].

## Methods

### In silico analysis

50 *E. coli* genomes available in GenBank® (http://ncbi.nlm.nih.gov/genbank/) were screened for presence of shiF and aerobactin using BLAST® algorithm (http://blast.ncbi.nlm.nih.gov/Blast.cgi)

### Bacterial strains and mutagenesis

S88 is an NMEC strain representative of clone O45:K1:H7 belonging to phylogenetic subgroup B2_1_ and harbours the large plasmid pS88 which contains a functional aerobactin operon [2]. Strain S88 was isolated in our laboratory. Its genome accession number is CU928161. We deleted *shiF* from S88 using Datsenko and Wanner’s method and the following primers Wanner-ShiF-1 (5′-ACAAATAAAC ACAATTAATACATGGTGTTGAAACATTACATGTGTAGGCTGGAGCTGCT-3′), Wanner-ShiF-2 (5′-CACAGGACGTTATGCCGGCTGTGAGTACAACATCATAGCC.

CATATGAATATCCTCCTTAG-3′) [9]. Two mutants, S88Δ*shiF1* and S88Δ*shiF2* were obtained by two distinct mutagenesis experiments. One hundred well-characterized and epidemiologically unrelated ExPEC strains were screened to assess the distribution of *shiF*. These strains have been previously described and isolated in France between 2007 and 2012 [2, 10, 11].

Other strains used in this study were: S88∆pS88 [2], *E coli* K-12 MG1655, *E coli* LG1522 [12] which is aerobactin dependent to grow under iron-limited conditions, the fully sequenced commensal *E. coli* strain ED1a and uropathogenic strain CFT073 which both harbor the aerobactin operon on their chromosome [13, 14]. Strains ED1-a, CFT073 and *E coli* K-12 MG1655 were obtained from Pr Erick Denamur head of our research team (Infection, Antimicrobials, Modelling, Evolution (IAME), University of Paris, France). Their genome accession numbers are respectively NC_011745, NC_004431 and NC_000913. Strain LG1522 was obtained from Pr Xavier Nassif (Institut Necker Enfants Malades (INEM) University of Paris, France).

### PCR prevalence of *shiF* in ExPEC strains

**Fig. 4:**
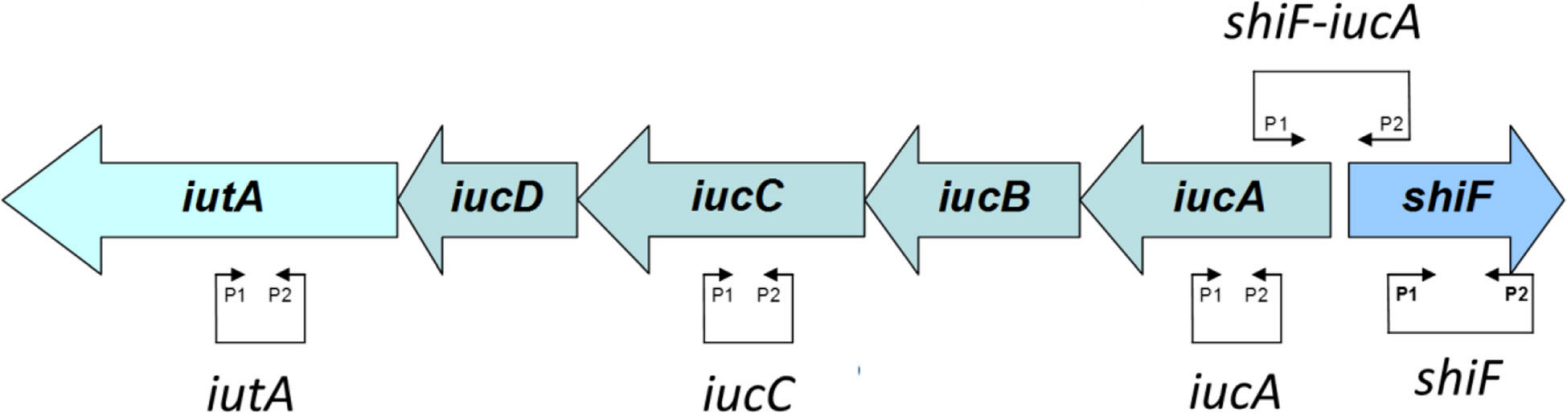
Schematic representation of aerobactin operon and *shiF*. The large arrows represent the genes of the aerobactin operon and *shiF IucA*, *iucB*, *iucC* and *iucD* encode the operon allowing aerobactin synthesis *and iutA* encodes aerobactin receptor. The small arrows annotated P1 and P2 represent the primers described in Table 1 and are located in front of their hybridizing sequences

The 100 ExPEC strains described above were screened by PCR to determine prevalence of *shiF* and its colocalization with aerobactin operon. Primers were based on the sequences of *shiF* and aerobactin operon in pS88 and are depicted in Fig. 4 and Table 1.

**Table 1:** Primers used for epidemiology of *shiF* and aerobactin

Name	5′-3′ Sequence	Gene	Product lenght
*shiF*-p.1	GATCGAAGATACGCCCCAA	*shiF*	1050 pb
*shiF*-p.2	CCATAGCCAAGTGTGTGACTG		
*iutA*-p.1	CCGTAACCCGGGCTGTAGTA	*iutA*	171 pb
*iutA*-p.2	ACCACCTCCTTTGACGTGAG		
*iucA*-p.1	AAGGGTCAACGATGGTGTTC	*iucA*	185 pb
*iucA*-p.2	ACAGCGAAGCGAATCTGATG		
*iucC*-p.1	TGGACGCTGAAACCTGGCTTACGCAACTGT	*iucC*	287 pb
*iucC*-p.2	CACGAAGTGACCCGTCTGCAAATCATGGAT		
*shiF-iucA*-p.1	TCACGAATCAAGGCATTCAG	*shiF/ iucA*^*a*^	400 pb
*shiF*-*iucA*-p.2	GATGGCAGAAACAGCATTGA		

^a^These primers are used to test the colocalization of *shiF* and *iucA* (Fig. 4)

All PCRs were performed with *Taq* DNA polymerase (Qiagen) on an iCycler thermal cycler (Bio-Rad, Marnes La Coquette, France) as previously described [4]. Positive controls were the strains S88 and CFT073.

### Cross-feeding assays

LB agar plate containing 200 μM of the iron chelator 2,2-dipyridyl, were seeded with strain *E. coli* LG1522 [12, 15]. The strains to be tested for aerobactin production were suspended in sterile water at an optical density at 600 nm (OD_600_) of 1 and then spotted onto the agar. Plates were observed after 24 h incubation at 37 °C for 24 h for growth of LG1522 strain around the spots.

### Growth assays

Strains were routinely grown in LB (Sigma) or in MM9 minimum medium (6 g Na_2_HPO_4_, 3 g KH_2_PO_4,_ 0.5 g NaCl, 1 g NH_4_Cl, 2 mM MgSO_4_, 0.1 mM CaCl_2_ per liter of water, pH 7.0) (Sigma-Aldrich) containing 20 mM glucose and 0.5% of an amino-acid solution (Vaminolact®, Fresenius Kabi, Belgium) in a rotary shaker at 37 °C. Strains grown for 18 h in LB at 37 °C with shaking were washed three times in fresh MM9 then diluted 1/10000 in fresh MM9 with 20 μM FeSO_4_ or 100 μM of 2,2′-dipyridyl to ensure iron limitation. Strains were then grown with shaking for 24 h at 37 °C in a multimode microplate reader (TECAN Infinite® M200 PRO) and OD_600_ was measured every 5 min was used to determine cell density. Calculated maximum growth rates, maximum cell density and lag were used for statistical comparison of the strains [16].

### Extraction and LC-MS/MS analysis of siderophores from culture supernatants and whole cells

Strains were suspended to an OD_600_ of 0.05 in 10 mL of MM9 with 100 μM of 2,2′-dipyridyl and grown for 14 h at 37 °C with shaking. After centrifugation, ferric chloride 0.1 M was added to cell supernatants at a final concentration of 3.75 mM, as previously described [17]. After 15 min at room temperature, the precipitate was removed by centrifugation (4000 g, 10 min). One milliliter of supernatant was evaporated to dryness under a gentle stream of nitrogen at 45 °C for 1 h. Dry residues were resolvated in 100 μL of 0.1% formic acid, and 5 μL was directly injected for analysis. For intracellular extraction, bacterial pellets from initial centrifugation were washed with ice-cold MM9 at + 4 °C, disrupted with 1.5 mL of ice-cold ethanol and sonication during 1 min and centrifuged again at 16000 g for 10 min [18]. One milliliter of the supernatants was then prepared as described above.

In order to obtain internal standards by isotopic labelling, strain S88 was grown in a rotary shaker for 14 h at 37 °C, in MM9 containing 20 mM of ^13^C-glucose as the sole carbon source and 100 μM of 2,2′-dipyridyl. ^13^C-labelled siderophores were extracted as described above and frozen for use as internal standards.

Siderophores were separated by reverse-phase liquid chromatography on a C18 column (Symmetry 2.1 × 50 mm, 3.5 μm, Waters) as previously described [4]. The liquid chromatograph (Separations module Alliance 2795, Waters) was coupled to a mass spectrometer with an *electrospray* ionization probe (Quattro micro™ Mass Spectrometer). Analyses were performed in positive ionization mode.

Strains to be compared were prepared together using same media, reagents and internal standard. The ^13^C-labeled internal standard was mixed to each culture supernatant (1:1) prior to siderophore extraction. Quantification was based on multiple reaction monitoring. Specific transition ions monitored from pseudomolecular ions to daughter ions used for detection and quantification of [M + H]^+^ aerobactin, [M + H]^+^ enterobactin, [M-2H + Fe(III)]^+^ ferric-yersiniabactin, [M + H]^+^ salmochelin S2 (source-fragmented) and [M + H]^+^ salmochelin S1 were 565 > 205, 670 > 224, 535 > 303, 506 > 319 and 627 > 224 *m/z*, respectively. The respective ^13^C-labeled isotopes were 587 > 213, 700 > 234, 556 > 314, 527 > 332 and 653 > 234 *m/z* [17]. Using MassLynx software, each siderophore was quantified by determining the ratio of the area under the peak to the area of its corresponding ^13^C-labeled internal standard.

### Statistical analysis

*P* values were calculated using Mann Whitney U-test for five to eight replicate per experiment. *P* values below 0.05 were considered statistically significant. Data are presented as mean ± standard deviation.

## Supplementary information

**Additional file 1: Figure S1.** Production of aerobactin analyzed by cross-feeding assay on LB agar plate containing 200 μM of 2,2-dipyridyl and seeded with strain *E. coli* LG1522. 1: S88; 2: S88∆shiF1; 3: S88∆pS88; 4: S88∆shiF2; 5: CFT073; 6: ED1a.

## Data Availability

All data generated or analysed during this study are included in this published article. The draft genome sequences of the 50 *E. coli* are available in GenBank® (http://ncbi.nlm.nih.gov/genbank/). *E. coli s*trains S88, ED1-a, CFT073 and K-12 MG1655 genome accession numbers are respectively CU928161, NC_011745, NC_004431 and NC_000913. The datasets used during the current study are available from the corresponding author upon request.

## Abbreviations

ExPEC: Extra-intestinal pathogenic *E. coli* strains
LB: Lysogeny broth
LC-MS/MS: Liquid chromatography-mass spectrometry
MFS: Major Facilitator Superfamily
NMEC: Neonatal meningitis *E. coli*
OD600: Optical density at 600 nm
ORF: Open reading frame
pS88: Plasmid carried by strain S88
