# Supplementary figures and images for: ShiF acts as an auxiliary factor of aerobactin secretion in meningitis Escherichia coli strain S88
Source: BMC Microbiol. 2019 Dec 17;19:298. doi: 10.1186/s12866-019-1677-2 (PMC6918656; doi:10.1186/s12866-019-1677-2)

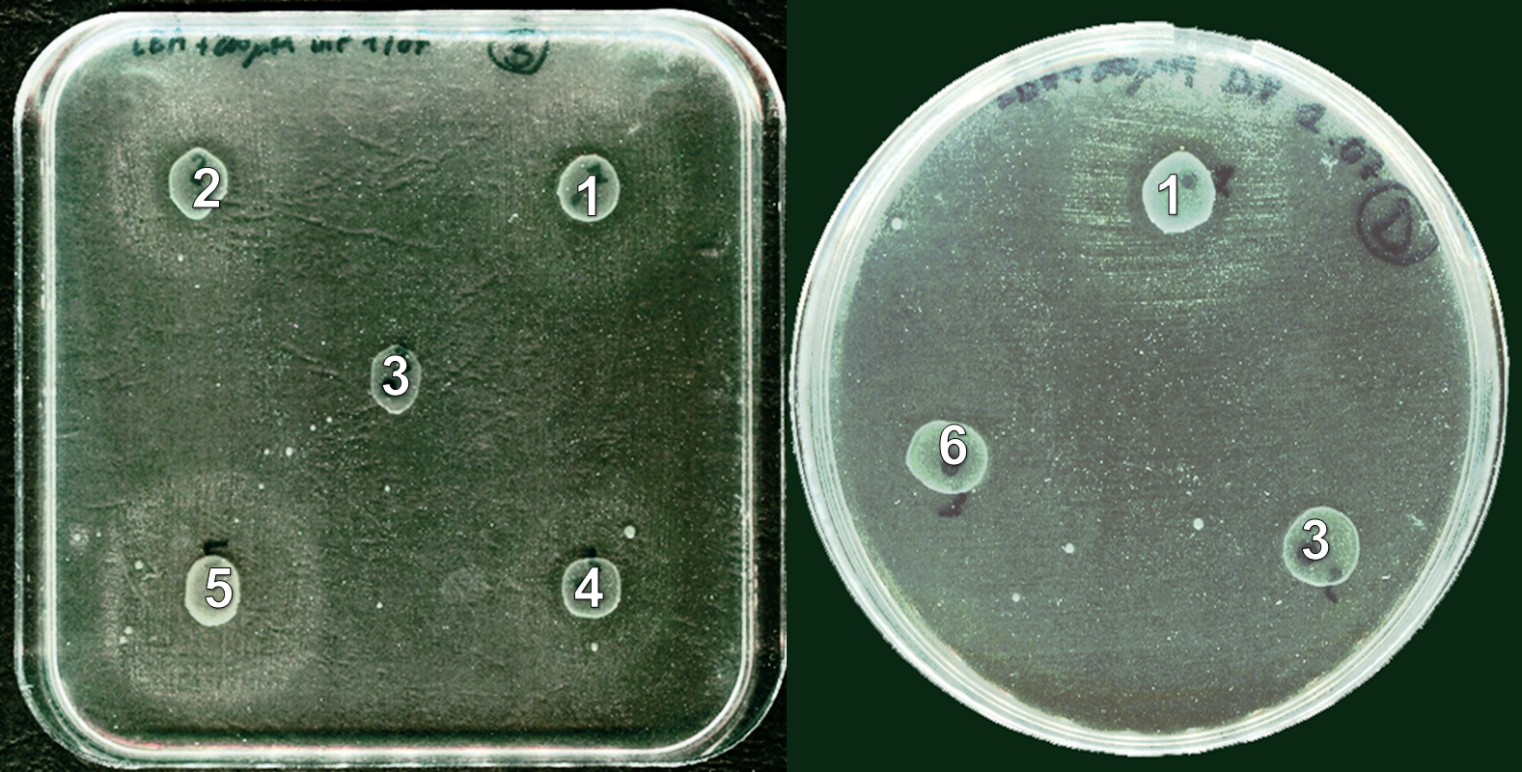

Supplement: Supplementary file 1 — Additional file 1: Figure S1. Production of aerobactin analyzed by cross-feeding assay on LB agar plate containing 200 μM of 2,2-dipyridyl and seeded with strain E. coli LG1522. 1: S88; 2: S88∆shiF1; 3: S88∆pS88; 4: S88∆shiF2; 5: CFT073; 6: ED1a. [file 12866_2019_1677_MOESM1_ESM.docx]
